# Underlying factors related to HIV/AIDS prevention: investigating the willingness to take pre-exposure prophylaxis among men-who-have-sex-with-men in Germany

**DOI:** 10.1186/s12981-021-00386-4

**Published:** 2021-09-17

**Authors:** Michele Pazzini, Zsófia S. Ignácz, Julia Tuppat

**Affiliations:** 1 Freie Universität Berlin (Alumnus), Berlin, Germany; 2 Goethe University Frankfurt, Frankfurt am Main, Germany; 3 University of Leipzig, Leipzig, Germany

**Keywords:** HIV pre-exposure prophylaxis, PrEP, HIV prevention technologies, Willingness, Men who have sex with men

## Abstract

**Background:**

This study investigates the willingness of men-who-have-sex-with-men (MSM) to use HIV pre-exposure prophylaxis (PrEP). Research in the HIV/AIDS field typically relies on clinical and epidemiological studies, thereby often excluding social dimensions of the illness as well as factors explaining its prevention. The current study analyzes HIV-prevention through an interdisciplinary theoretical approach. It aims to comprehensively understand the mechanisms associated with the willingness to take PrEP among MSM in terms of psychological, social, behavioral, cultural, and demographic factors.

**Methods:**

We analyze data from the survey “Gay Men and AIDS” conducted in Germany in 2013 prior to market approval for PrEP. Analyses were performed using the statistical software SPSS 25.0, while results were visualized using the R programming language.

**Results:**

We find that perceived risk of infection, social norms (anticipated HIV-stigma), practices (e.g. regular condomless sex), and socio-demographic factors (young age, being single) all have a positive effect on the willingness to take PrEP, while education reveals a negative, and income no effect.

**Conclusions:**

Results indicate that beyond well-established socio-psychological mechanisms of health behavior, social factors play a crucial role in understanding the willingness of PrEP uptake. This study enriches existing health behavior theories with sociological concepts such as social norms and social practices.

## Background

HIV researchers, policymakers, and other stakeholders have targeted 2030 as the year to end the HIV epidemic [1]. Prevention technologies are expected to play a significant role in this success [2]. Among strategies that stop new HIV infections, pre-exposure prophylaxis (PrEP) is the most innovative tool [3]. PrEP is a combination pill of emtricitabine and tenofovir [4], two drugs also included in the antiretroviral therapy (ART) that suppresses the viral load in HIV-positive people [5].

North American and European clinical trials demonstrated the efficacy of PrEP: it reduces the risk of HIV infection by 86 to 92% [6–8]. The World Health Organization [9] and the U.S. Centers for Disease Control and Prevention (CDC) recommended the European Union to integrate PrEP into its preventive packages for high-risk subpopulations, prioritizing men who have sex with men (MSM). This group constitutes the majority of people living with HIV in Western countries [10]. In 2013, for example, MSM represented 42% of the HIV diagnoses in Europe [6]. The same trend has been observed for Germany [4, 11, 12]. Although MSM is one of the populations most vulnerable to HIV, social group identity is not considered a proxy for HIV acquisition. The risk of infection is associated with behavior, not sexual orientation, as displayed by the CDC’s 2013 statement that condomless anal intercourse carries the highest risk of HIV acquisition [13]. While the clinical utility of PrEP is clear, populations most exposed to HIV must be willing to engage in preventions for PrEP to become an effective tool against HIV.

Flowers [14] identified three phases in the history of the HIV/AIDS epidemic. Each phase was linked to specific risk-reduction strategies: (1) the reduction of risky sexual behavior (1981–1986), (2) the recommendation of wearing a condom for every sexual encounter, and (3) since 1996, the administration of ART to infected people transforming HIV from a progressive illness with a fatal outcome into a manageable chronic disease [15]. Likewise, this article aims to position PrEP as a further phase in the history of HIV because PrEP has the potential to redraw landscapes of risk during sexual encounters [5, 16]. In fact, as shown by clinical trials condomless sex is no longer a great HIV risk when coupled with PrEP use [6, 7].

This study investigates the willingness of MSM to take PrEP by analyzing data from the cross-sectional survey “Gay Men and AIDS” (originally “Schwule Männer und AIDS”) fielded among MSM living in Germany in 2013 before the European Medicines Agency approved PrEP for the market [8]. In 2016, PrEP became accessible in Germany with a medical prescription, although it was not subsidized by state health insurances. As of September 2019, PrEP and the related checks for sexually transmitted infections (STIs) are covered by statutory health insurances [17]. The German guidelines recommend PrEP for high-risk MSM reporting condomless anal intercourse in the last three to six months and/or a with curable STI in the last year, and for MSM with a HIV-positive partner not taking ART [18].

Research in the HIV/AIDS field typically relies on epidemiological and clinical studies, which often exclude social dimensions of the illness [19, 20]. Studies on motivators for PrEP usage are mostly based on theories of behavioral change [13] that apply individualistic approaches [21]. However, with reference to the interplay between agency and structure, social scientists debate the necessity of also analyzing social determinants and cultural dimensions, in which individual behavior is embedded [13, 22, 23]. For instance, social researchers of HIV/AIDS emphasized the role of networks in shaping sexual norms, assuming a decreased likelihood of condomless intercourse when MSM perceive their peers to be supportive of risk-reduction norms [24].

Relying on work from diverse fields [5, 16, 25], this study applies an interdisciplinary approach to comprehensively understand what motivates PrEP uptake in terms of psychological, social, behavioral, cultural and demographic factors. The approach enriches cognitive frameworks, such as the Theory of Planned Behavior [26–28] with sociological concepts of social norms and practices to explain the underlying mechanisms influencing the willingness to take PrEP among MSM living in Germany.

Previous research found the acceptability of PrEP among MSM positively associated with risky practices like condomless intercourse [3, 29] combined with recreational drug use [4]. Sagaon-Teyssier and colleagues [30] identified associations between interest in PrEP and age, relationship status, educational level, and subjective high-risk of contracting HIV.

PrEP acceptability and willingness among MSM have been primarily investigated by recruiting participants from identified gay areas in major cities, for instance New York [31–33], Chicago [24], Amsterdam [34], London [35] and Berlin [8]. Despite the availability of extensive data about PrEP adherence and efficacy from European randomized controlled trials [6, 7] there is limited capacity of quantitative analyses about the relationship between social norms, sexual practices, and risk-reduction strategies such as PrEP [5, 16, 24]. This study intends to fill those gaps, investigating data from a nationwide survey conducted in Germany and combining individual-level measures with wide socio-cultural constructs [25, 36, 37].

## Towards a multidisciplinary understanding of HIV-prevention

Most studies on motivators for PrEP usage are based on theories on behavioral change derived from social psychology. For instance, the widely applied Health Belief Model [38] states that individuals’ beliefs about health problems, perceived benefits, barriers to action, and self-efficacy are key in explaining health-related behavior. Likewise, the Theory of Reasoned Action [39] assumes the best predictor of behavior is intention, which is determined by attitudes and social normative perceptions towards the behavior. The subsequent Theory of Planned Behavior [26] adds behavioral control as a further mechanism. While these theories provide some background for understanding the motivators of PrEP usage, they are still incomplete. Overall, the role of rationalized individuality is overemphasized in these theories [13].

**Fig. 1:**
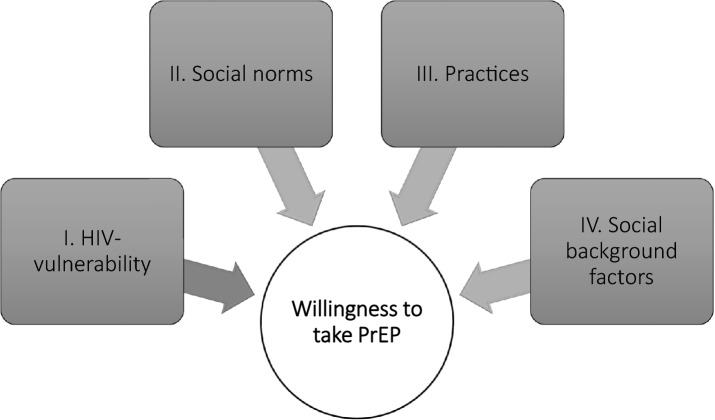
Theoretical model

This study presents an integrated model of the willingness to take PrEP, which incorporates the different theoretical approaches outlined above. The model explains which determinants affect the willingness to take PrEP among MSM living in Germany and integrates explanations from epidemiological, psychological, and sociological research [31, 32, 34, 40–44]. Figure 1 depicts the theoretical model, which focuses on the synergistic effect of four explanatory areas: (I) HIV-vulnerability, (II) social norms, (III) practices, and (IV) social background factors.

### Explanatory areas

I. HIV-vulnerability*.* The first key mechanism is the perception that one is at risk to be infected. For instance, the Risk-Reduction Model developed by Catania et al. [45] focuses on psychological factors that make people perceive their sexual acts as problematic. Identifying their high-risk behaviors, individuals begin to feel vulnerable toward HIV infection. HIV-vulnerability refers to beliefs about exposure [46, 47]. Perceptions of the risk of infection positively impact the willingness to use anti-HIV strategies [48]. Previous studies found MSM who perceived themselves more likely to become infected with HIV to be more willing to use PrEP [3, 32, 34]. Accordingly, we assume that increasing HIV-vulnerability is associated with a greater willingness to take PrEP (hypothesis 1).
II. Social norms. Social norms impact the labeling of health problems through disapproval of high-risk activities and encouragement of safe behaviors [45]. The Reasoned Action Theory [28] conceptualizes subjective norms as cognitive functions that stem from the perceived social pressure of one’s environment, and/or support from peers. Accordingly, relationships with friends, sexual partners, and family members may strongly influence an individual’s choice to engage in risk-reduction behaviors [24, 46]. This study addresses the notion of anticipated HIV-stigma, which reflects individuals’ fear of being rejected by others [49], as well as perception of what peers or family members may think or how they might react if subjects became infected with HIV. The concept thereby refers to the internalization of negative societal attitudes toward HIV-positive people [50]. Anticipated HIV-stigma has been demonstrated to operate as a concern exacerbated prevalently among those MSM displaying greater risk perceptions of HIV infection [49]. Thus, we expect that higher anticipation of HIV-stigma is associated with a greater willingness to take PrEP (hypothesis 2).
III. Practices. By replacing the term sexual behaviors with that of practices, we emphasize an understanding of human behavior with reference to its social production and cultural patterns [25]. Sexual practices are influenced by the physical context in which decision-making between partners occurs, i.e. the association between a given environment and the safe or risky sexual practices enacted within it [51]. Here, the focus is on practices that have been proven to increase the risk of HIV transmission, namely (a) attendance of sex-on-premise venues [35], (b) e-dating [52, 53], (c) condomless casual intercourse [51], and (d) drug use [54–56]. Our expectation is that those engaged in riskier practices still want to avoid becoming infected with HIV, and therefore, more likely are willing to take PrEP.
  - Attendance of sex-on-premise venues. Aghaizu et al. [35] considered the commercial sex-on-premise venues as spaces that increase chances of HIV infection among MSM. Affiliation with saunas, dark rooms, and sex clubs was found to be associated with high exposure to HIV transmission [57, 58] and growth of HIV diagnoses [51]. We assume increased attendance of MSM sex-on-premise venues to be associated with greater willingness to take PrEP (hypothesis 3a).
  - E-dating. Over the past decade, using the Internet to find sexual partners has increasingly become a new risk environment. Contacting casual partners through websites or smartphone applications is not risky per se. However, it allows individuals to meet more sex partners, thereby increasing exposure to HIV [52, 57, 59]. We assume that increasing frequency of casual sex partners met through e-dating platforms is associated with greater willingness to take PrEP (hypothesis 3b).
  - Condomless intercourse. Condomless anal sex is the primary high-risk behavior responsible for HIV-transmission [60]. Despite the widespread recommendation to always wear condoms, a significant proportion of MSM continue to engage in condomless sex [2, 14]. Condoms may represent a barrier to intimacy in both steady relationships and casual encounters [41]. MSM who attribute great losses of benefits to condom use may consider alternative safe strategies [48] such as PrEP. This assumption has been supported in several studies [3, 32, 35, 42, 61]. Accordingly, we assume that more engagement in condomless intercourse is associated with greater willingness to take PrEP (hypothesis 3c).
  - Drug use. Physical energy, feelings of connectedness, disinhibition, and sex drive can be stimulated by using recreational drugs at dance club parties [54, 56]. Sildenafil (Viagra® in commercial use) was found to be associated with condomless sex either used alone or in combination with other substances [52, 56]. Furthermore, consumption of club drugs was found to be positively related to PrEP interest [32, 62]. We expect the use of club drugs and/or sildenafil to be associated with greater willingness to take PrEP (hypothesis 3d).
IV. Social background factors. Cognitive theories of health behavior change posit that non-motivational elements, such as personal and socio-demographic characteristics, contribute to explaining the behavioral choices with regard to HIV prevention [27, 63]. This study analyses the effects of (a) age, (b) relationship status, (c) educational attainment, and (d) monthly net income.
  - Age. Previous studies consistently found that younger respondents displayed greater willingness to take PrEP [61, 64–66]. However, mixed results have been reported in studies measuring age in association with actual use of PrEP: Several studies reported that PrEP users were more likely to be of older age [67, 68] while other studies reported a greater likelihood of usage among the younger MSM [69, 70]. For its research purpose, which investigates the willingness to take PrEP, the current study will examine age in accordance with previous findings indicating higher willingness to take PrEP among younger MSM. On the one hand, this association may reflect an age dependency of specific social practices, such as risk behaviors. On the other hand, the effect of age might be related to the history of the HIV epidemic and may instead reflect a cohort effect rather than an actual age effect. For instance, according to Grov et al. [42], MSM who came of age since the launch of ART to treat HIV infections have not been the target group of widespread community-based recommendations of wearing a condom for every sexual intercourse in the early HIV phases, unlike older cohorts [14]. We therefore assume older MSM to be less willing to take PrEP (hypothesis 4a).
  - Relationship status. MSM without a steady relationship demonstrated more interest in using PrEP in previous studies [33, 34, 61]. Single MSM have more opportunities to engage in casual sex than MSM with steady partners; consequently, their exposure to HIV is higher. We expect single MSM to display greater willingness to take PrEP than those in steady relationships (hypothesis 4b).
  - Educational attainment. Previous studies did not identify a consistent association between education and PrEP willingness. Some studies reported a lack of significant association [32, 33], others showed that MSM possessing less than a high school degree display greater intention and likelihood to use PrEP [42, 61, 64] than those who those who achieved a more higher level of education [70, 71]. Yet, when it comes to actual PrEP uptake, the association between level of education and PrEP use seems to be reversed: having a university education was found to be associated with PrEP use [66, 68, 72]. The present study examined data surveyed prior to the implementation of PrEP in Germany, where access to information was already available, but experience was possible only through informal resources other than the healthcare system; therefore, we build our hypothesis on previous research on willingness and not actual PrEP uptake. When commenting on the surprising negative association between education and PrEP willingness, which is contrary to the findings on PrEP uptake, Grov et al. [42] speculate that this might be due to greater health literacy among the more educated. The authors argue that those more informed might contemplate the potential side effects more, leading to less willingness towards PrEP. Thus, with consideration to the general climate related to PrEP at the time of the survey, before encouraging results about the safeness of PrEP from the PROUD and Ipergay projects became public [6, 7, 73], we assume that greater knowledge of PrEP regime and its potential side effects may have increased concerns about taking PrEP and therefore, higher educated MSM could have been more skeptical toward PrEP in Germany in 2013.We therefore, expect that MSM with lower levels of education display greater willingness toward PrEP than those with higher level of formal education (hypothesis 4c).
  - Net income level. Previous research found a negative association between income and openness towards PrEP [3, 62]. A recent investigation in France under circumstances where PrEP was already accessible through the healthcare system reported higher intention to take PrEP among low-income groups, such as unemployed and student MSM [64]. Correspondingly, findings focusing on PrEP uptake show that financial means play an important part. For instance, studies conducted in Germany when PrEP was already accessible through the healthcare system for monthly payment of 50–70 EUR and without routinely medical examinations covered by statutory insurances showed that MSM rather purchased PrEP through informal and cheaper sources than the legal market due to unaffordable costs via official providers [17, 69], supporting the notion of higher willingness among MSM with lower income. Accordingly, we expect a negative association between income and willingness (hypothesis 4d).

## Methods

### Data

The investigation of MSM’s willingness to take PrEP is based on the German-wide online survey “Gay Men and AIDS” [74]. It was funded by the German Federal Centre for Health Education, and approved by the ethical review board of the Charité University Clinic in Berlin [11]. Participants were recruited through social networking and dating websites predominantly accessed by MSM [75].

Compared to personal sampling techniques, online sampling has the advantage of reaching community members who wish to remain anonymous while responding to sensitive questions about their sexual practices and medical background [76]. Of course, known problems about nonprobability sampling regarding representativeness and cautious generalizability exist for this dataset as well [77].

An important feature of the survey is that it was completed in 2013 prior to the WHO’s recommendations and the introduction of PrEP into the German healthcare system. This survey also has the advantage that it allows to filter respondents for their knowledge about PrEP explicitly. Thus, we can identify influencing factors for the willingness to take PrEP among MSM in a clear manner, so that the assessment of influencing factors on PrEP willingness is not jeopardized by lack of awareness of, and familiarity with, this medicine.

### Variables

The dependent variable was the respondents’ willingness to take PrEP, which was assessed with the following question: “In principle, would you be prepared to take a medication as a preventive measure to reduce the risk of HIV infection?” During the survey, respondents were adequately informed about PrEP (see details in Appendix, Table 1 about information provided). Their answers were recoded into a dichotomous variable (0: ‘No’ and 1:’Yes’).

Several explanatory variables were included in the analysis (for exact wording of the items see Appendix Table 1).

I. Subjective HIV-vulnerability. The survey queried the respondents’ perception of their risk of contracting HIV in the last 12 months on an 11-point scale (0: ‘No risk’ to 10: ‘High risk’).
II. Anticipated HIV-stigma. The extent to which participants anticipated negative consequences if they were to seroconvert in the future was measured to quantify the relevance of social norms [49, 61]. To measure anticipated HIV-stigma we created a mean index from four items on the basis of factor analysis and an internal consistency measure (Cronbach’s alpha = 0.86). The items refer to anticipated negative social consequences in case of an HIV infection (“My family would be disappointed”, “Friends would reproach me”, “Friends and acquaintances might think I’ve failed”, “Family and friends would avoid me”). The index measured anticipated HIV-stigma ranging from 1: ‘Very unlikely’ to 4: ‘Very likely to occur’.
III. Practices. Engagement in high-risk practices was operationalized by several variables.
  Attendance of sex-on-premise venue. Based on results from factor analysis, attendance at risky venues (i.e. dark rooms, sex-clubs, public and private sex-parties, saunas and pornographic cinemas, and outdoor cruising areas) were captured in a composite index (Cronbach’s alpha = 0.76), which measures the frequency of attendance in such venues from 1: ‘Never’ to 5: ‘Very frequent’.
  Engagement in e-dating was operationalized by the proportion of the respondents’ casual sex partners met in the last year through online platforms on a 6-point scale from 0: ‘None’ to 5: ‘All of them’.
  Condomless intercourse. The frequency of using condoms (or lack thereof) during casual anal intercourse in the last 12 months that respondents reported, was measured ranging from 1: ‘Always (uses condoms)’ to 5: ‘Never (uses condoms)’.
  Drug use. Respondents were asked if they have used any of the following recreational drugs more than twice in the last year: methylenedioxy-methamphetamine (or ecstasy), amphetamine, marijuana, mephedrone, cocaine, heroin, gamma hydroxybutrate (GHB) or ketamine. The same dichotomous measurement was applied to the use of sildenafil (Viagra®). We combined the two variables into a typology to capture drug use practices (1: ‘None’, 2: ‘Party drugs only’, 3: ‘Viagra only’, and 4: ‘Both’).
IV. Social background factors. Individual characteristics of respondents were operationalized by the following variables: age (measured in years), relationship status (0: ‘Not single’ and 1: ‘Single’), educational attainment (0: ‘Lower secondary level or below’ and 1: ‘Upper secondary level or higher’), and monthly net household income measured in quintiles (1: ‘Lowest quintile’ to 5: ‘Highest quintile’). The categorical information on household income was not equivalized for household size, as information on the actual net-equivalized household income was available for a limited number of respondents only. Two robustness checks were conducted: (1) the analysis was repeated using information on the net-equivalized household income with the restricted sample, which did not change the direction of the effect, and (2) a correlation analysis was conducted between the household income categories and the net-equivalized household income (r = 0.8). We can confidently state that using the income categories most likely did not introduce bias into our analyses and substantially increased the sample size.

### Description of analysis

A series of logistic regression analyses were used to reveal the effect of the explanatory variables on the willingness to take PrEP. Sets of independent variables (corresponding with the explanatory areas) were individually analyzed to assess their contribution to the model. The final logistic regression analysis is the full model including all of the independent variables. Thus, the paper tests five models. In the first four models presented in the Results section, only variables corresponding to a specific explanatory area are included in the analysis. In the final model, all variables from all of the explanatory areas are included to account for, and the odd ratios (ORs) reported are from the multivariate logistics regression. In none of the models was there indication of multicollinearity among the independent variables. The analyses focus on the explanatory power of the coefficients derived from the logistic regression models, so the ORs are reported (instead of logged odds [78]). ORs indicate the likelihood of a respondent willing to take PrEP relative to the likelihood of rejecting that notion. While OR values from multiple models cannot be accurately compared in their absolute sense, their level of significance and the general tendency are comparable. Therefore, we depict the ORs on the same figures in the “Results” section [79].

The statistical software SPSS 25.0 was used to perform all the analyses, while the results were visualized using the R programming language.

## Results

After listwise deletion, the sample consisted of 2948 MSM. While 16,734 MSM had initially been recruited for the survey, a large proportion of the initial sample was dropped from the analysis. These were respondents who reported HIV-positive serostatus and so did not answer PrEP-related questions (N = 1437), who stated that they are sexually attracted to women only (N = 26), who stated limited knowledge about PrEP to express their willingness to take it or not (N = 5969), and who refused to answer the question on condom use with casual partners, a key variable of our analysis (N = 6945). A robustness check without the variable condom use was also conducted to acquire a larger sample size (N = 4914). This did not change the main effects of the remaining explanatory variables (see Appendix Fig. 4 for the results when the variable was excluded).

**Fig. 2:**
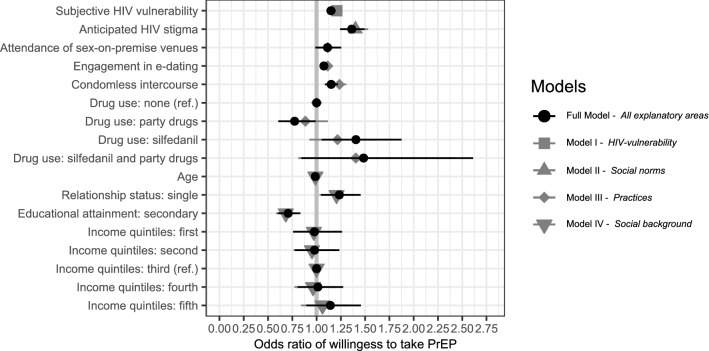
Odds ratio of willingness to take PrEP according to the explanatory areas. Variables included: subjective HIV-vulnerability, anticipated HIV-stigma, attendance of sex-on-premise venue, engagement in e-dating, condomless intercourse, drug use, age, relationship status, educational attainment, monthly net household income; All variables are unstandardized, N = 2948

Figure 2 summarizes the ORs. The primary focus is on the full model and its ORs, as this contains information about all of the variables corresponding to the identified explanatory areas.

On average, the odds of a participant’s willingness to use PrEP increase, the higher the risk of becoming HIV-positive is assessed. This supports hypothesis 1. The odds of being willing to take PrEP increased across the sample if respondents felt that they were likely to contract HIV in the previous year (OR: 1.15, CI: 1.10–1.20). This result explains that the probability of willingness to take PrEP was 1.15 times higher for an additional unit of increase in perceived risk of HIV-infection. In other words, with a one-unit increase in the perceived risk of HIV-infection, the odds of being willing to take PrEP increase by 15%.

The second explanatory area is the social norms related to HIV. The findings displayed a significant and positive effect of anticipated HIV-stigma on the outcome (OR: 1.37, CI: 1.24–1.49). More precisely, for each increasing unit in scores of anticipated HIV-stigma within the sample, the willingness to take PrEP rose by 37%, supporting hypothesis 2. This rather strong effect indicates that the role of social norms is essential in understanding individual behavior.

The third explanatory area refers to different practices—primarily involving risky sexual behavior—that contribute to HIV-exposure among MSM. All of the variables associated with this area explain the willingness to take PrEP significantly or somewhat significantly. In line with hypothesis 3a, attending sex-on-premise venues, where contexts like saunas, sex clubs, and dark rooms more likely enable casual sex with one or multiple partners, marginally increased the likelihood that a respondent was willing to take PrEP (OR: 1.11, CI: 0.99–1.25). Engaging in sexual activities through e-dating indicated positive and significant associations with willingness in support of hypothesis 3b: involvement in e-dating increased the willingness to take PrEP by 8% (OR: 1.08, CI: 1.03–1.12).

The frequency of condomless intercourse was also associated with the outcome as expected by hypothesis 3c. The less regularly respondents use condoms during casual anal intercourse, the more likely they are willing to take PrEP (OR: 1.15, CI: 1.09–1.22).

Lastly, a moderate association can be seen between drug use and willingness to take PrEP. The direction of the effects is opposite for party drugs and for sildenafil. While taking club drugs, such as amphetamine, GHB and ketamine, reduces the willingness of an MSM to take PrEP compared to those who refrain from consuming recreational substances (OR: 0.774, CI: 0.61–0.99), willingness increases among respondents who regularly use sildenafil (OR: 1.41,CI: 1.05–1.88). This only partially supports hypothesis 3d. Overall, respondents from the sample who practice risky sexual behaviors exhibit an increased willingness to take PrEP, except for those who regularly take party drugs.

The final set of model variables reflects socio-demographic factors. In support of hypothesis 4a, the age of respondents displayed a significant negative association with willingness of PrEP intake (OR: 0.99, CI: 0.98–0.99) indicating that the odds of being willing decreased by 1% for each additional year of age. Accordingly, a 10-year increase in respondents’ age lowered their willingness to take PrEP by 9.6% [(0.99^10^) = (0.904–1) • 100 = − 9.6]. In support of hypothesis 4b regarding relationship status, being single increased respondents’ willingness to take PrEP by 23.6% (OR: 1.23, CI: 1.05–1.45) compared to those MSM in a relationship.

The outcome variable was also significantly associated with educational attainment. Respondents who finished secondary school or higher have lower odds of willingness to take PrEP by around 29% (OR: 0.71, CI: 0.60–0.83).

Income is the only characteristic that did now show a significant association with willingness to take PrEP. Although the direction of the correlation is in line with our expectations, hypothesis 4d cannot be supported based on our model. Income does not significantly decrease the willingness to take PrEP for the MSM in our sample.

## Discussion

This paper analyzed the willingness to take PrEP among MSM in Germany. The primary goal was to develop a comprehensive understanding of the mechanisms associated with willingness to use PrEP in terms of psychological, social, behavioral, cultural and demographic factors. This approach enriches existing theories of health behavior with sociological concepts such as social norms and social practices in order to explain the underlying mechanisms that influence MSM’s willingness to take PrEP. Four explanatory areas were investigated: perceived risk of infection (HIV-vulnerability), social norms (anticipated HIV-stigma), practices, and demographic factors.

Nearly every explanatory factor yielded significant effects on the willingness to take PrEP. Factors from all explanatory areas were significantly associated with the outcome variable. More importantly, factors from all four explanatory areas remained relevant, even when controlled for with factors from other explanatory areas.

From the explanatory areas, subjective HIV-vulnerability and social norms were most strongly correlated with willingness to use PrEP. Socio-demographic factors proved to be relevant, yet they had an overall weaker effect. The analysis corroborates that increased HIV-vulnerability is correlated with greater willingness to take PrEP (hypothesis 1), as are increased anticipation of HIV stigma (hypothesis 2), attendance of MSM sex-on-premise venues (hypothesis 3a), increased frequency of casual sex partners met on e-dating platforms (hypothesis 3b), more frequent engagement in condomless intercourse (hypothesis 3c), use of club drugs and/or sildenafil (hypothesis 3d in part) and being single (hypothesis 4b). The finding that being single increases the willingness to take PrEP should be interpreted with caution, however, because every non-single respondent is not necessarily in a monogamous relationship; members of open relationships agree to have sexual encounters with partners outside of the relationship [22]. In contrast, MSM who take party drugs (hypothesis 3d in part) and are older (hypothesis 4a) and have attained at least secondary education or higher (hypothesis 4c) were found to exhibit more reluctance to take PrEP.

Our findings are consistent with results from other studies. The confirmation of hypothesis 1 about risky sexual behavior mirrors previous findings [32, 33, 48]. In particular, Bauermeister et al. [48], found that estimates of risk affected the likelihood to use HIV-prevention strategies. Similarly, data surveyed among MSM in Germany in 2016 [65], and in Berlin between 2017 and 2018 [29], when PrEP was already accessible through the healthcare system but not yet covered by the insurance (statutory health insurance covers PrEP since 2019 in Germany), also show that the self-perception of having been at-risk of HIV-acquisition among the respondents increase chances of PrEP uptake. The current study confirmed the essential role of social norms in understanding individual behavior, as reported in previous studies [24, 49], by applying an index for anticipated HIV-stigma to measure the relevance of social norms on the willingness to take PrEP.

Furthermore, the current study also found significant association between different practices that potentially increase HIV-exposure among MSM and their willingness towards PrEP uptake. Consistent with previous research, the increased attendance of MSM sex-on-premise venue means higher willingness to use HIV-prevention strategies [32, 42, 61], as does more engagement in sexual encounters through e-dating platforms [57, 58]. Moreover, findings confirmed that the less regularly respondents used condoms during casual anal intercourse, the more willing they were to take PrEP. These results mirror data surveyed in 2018 reporting that since taking PrEP half of the analyzed MSM living in Germany indicated using condom fewer times than before, while 21.3% stopped using condom completely [17]. This correlation is very meaningful, considering that condomless anal sex increases both the chance of acquiring and transmitting the virus, in particular when subjects ignore their HIV-positive serostatuses [80].

Results from this study confirmed increased willingness to take PrEP among the younger and less educated MSM, echoing research with related findings [61, 64]. Findings corroborate Grov et al. [42], who found MSM with less than a high school degree more interested in taking PrEP because they could be less familiar with its characteristics. In fact, the potential side effects and compelling requirements to begin and adhere to PrEP regimen have been assumed to be aspects that discourage people from taking it [81].

**Fig. 4:**
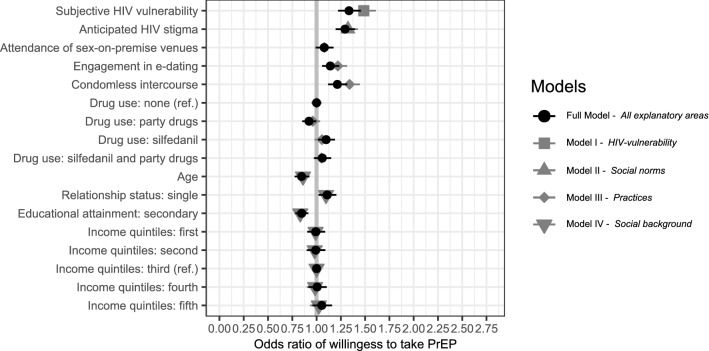
Odds ratio of willingness to take PrEP according to the explanatory areas (Z-standardized independent variables). Variables included: subjective HIV-vulnerability, anticipated HIV-stigma, attendance of sex-on-premise venue, engagement in e-dating, condomless intercourse, drug use, age, relationship status, educational attainment, monthly net household income; All variables are Z-standardized, N = 2948

The online survey “Gay Men and AIDS” is one of the richest and most in-depth secondary data sources available on MSM living in Germany. It provides researchers with wide-ranging additional information about respondents beyond the topic of HIV. However, it is also marked by several drawbacks stemming from its online sampling technique, large number of non-response for some items, the fact that it is a secondary data source, and the time of the field survey. One of the survey’s largest drawbacks is its lack of representativeness, which stems from it being conducted online. The sample underrepresents those from lower income levels, and those with lower education levels. Access to the older population is also challenging for online surveys and this is also reflected in the demography of the current study. Thus, it remains unclear, whether the results can be generalized for the whole population. Furthermore, the online format and the delicate topic at-hand resulted in large number of missing information for several crucial items. In particular, a disproportionally high rate of respondents refused to disclose whether they engage in condomless intercourse, and the analysis lost many valid cases. Overall, however, we deem this only of a minor issue, because through robustness checks we can show that the identified associations are persistent and comparable even when we include all respondents, who refused to disclose their habits about condomless intercourse (see Appendix, Fig. 4). Additionally, the definition of some factors (operationalization), particularly the social background factors, is less than ideal due to the survey being a secondary data source. The authors must also concede that especially the operationalization for age is limited. There are no means to effectively disentangle the cohort and age effects from one another, as the survey is cross-sectional. Should cohort effects be the main drivers of the association we see between age and willingness to take PrEP, as per the argument of Grov and colleagues [42], we should see a weakening association between age and willingness in studies conducted several years later. An ideal means to disentangle these two effects from each other is the implementation of longitudinal survey on the topic.

Finally, another crucial limitation of the survey is that it was conducted in 2013. This is before PrEP became (legally) accessible in Germany, and before results of high quality trials for administering PrEP was available [6, 7, 73]. In the light of this, we can expect that the knowledge about PrEP of MSM in Germany has increased in the past years (however, we can only speculate how much the knowledge about PrEP has actually increased, since no repeated time data is accessible to trace the trend in knowledge increase over the past years). From this follows, that had the survey been conducted in later years, more respondents would have reported sufficient knowledge about PrEP and would have ensured the current study with a larger sample size. However, when considering the demography of those with and without sufficient knowledge of PrEP in the survey, data shows that there is very little difference between the two (see Appendix, Tables 3, 4, 5). The temporal validity also plays to the strength of the paper and its findings, since the survey was conducted in Germany only shortly after the Food and Drugs Administration’s approval of PrEP in 2012 for persons at high-risk of HIV-infection in the U.S. Besides, only off-label use of PrEP (purchased through informal sources) was available in Germany at the time the survey was conducted [82]. Such circumstances ensure a methodological clean research design about how the (future) willingness of the uptake of PrEP depends on (past and current) values, attitudes, and practices. This means that all respondents are largely on equal grounds with their experience with PrEP. Their willingness to take PrEP is not muddled with previous first-hand (or second-hand) experience with PrEP nor their past behavior related to the uptake of PrEP. Likewise, in contrast to actual PrEP uptake, the willingness to take PrEP is neither dependent on external constraints (e.g., costs), nor potential external pressures to take PrEP (e.g., by partners or spouses), but expresses the pure motivation of MSM to use PrEP. Nonetheless, later research based on surveys conducted since PrEP’s rollout in Germany should investigate how circumstances, attitudes, willingness, and behavior to use PrEP have changed since the survey in 2013. For this purpose, the present study may serve as a benchmark for future research.

## Conclusions

This paper shows that willingness to take PrEP relates to several interpersonal and societal factors. Thus, it is crucial to expand discussions about PrEP beyond the epidemiological and medical dimensions and incorporate social aspects. Furthermore, the paper demonstrates how the Theory of Planned Behavior can be applied to this topic as well and systematically brings various factors together under a unified theoretical framework for the first time. Thus, the mechanisms identified in this study for the willingness to take PrEP can help aid future studies pinpoint the most crucial factors that influence behavior and the actual uptake of PrEP.

Moreover, the findings of the paper have wide-reaching implications. First, identifying several interpersonal and societal factors that influence the willingness to take PrEP means that the success and widespread use of PrEP depends on more than the health care institutions and the medical assistance the health care system can provide to make it available to the MSM community. Much is dependent on individual habits and attitudes, which are embedded in the social environment of potential users beyond. Second, this theoretical impact on the societal dimensions relevant for PrEP, this paper potentially foreshadows developments in the HIV epidemic as PrEP has undoubtedly opened a new chapter in dealing with HIV. However, the current accessibility to PrEP is not widespread globally enough for it to achieve the goal of eradicating HIV by 2030. In fact, although in European and Central Asian countries progress has been made between 2016 and October 2020, with 16 out of 53 countries providing and reimbursing PrEP within their national healthcare system, PrEP implementation across those countries remains fragmented and complex. To accelerate the progress toward the end of the HIV epidemic by 2030 it is important that knowledge about PrEP should be shared between countries [83]. Thus, the current study can serve well policy makers involved in public health interventions and medical practitioners active in countries falling a few steps behind the German policy developments. Furthermore, the study has considered aspects that go beyond the individual risk-taking represented by condomless sex, and has identified important characteristics of MSM. Such knowledge supports medical practitioners not only to identify potential PrEP-users, but also those MSM more apprehensive about using PrEP. And the study’s findings can also support the development of further HIV-prevention strategies. Thus, results from this study can support community-based organizations and groups to advocate for PrEP in countries where its implementation has not yet occurred.

## Figures and Tables

**Fig. 3:**
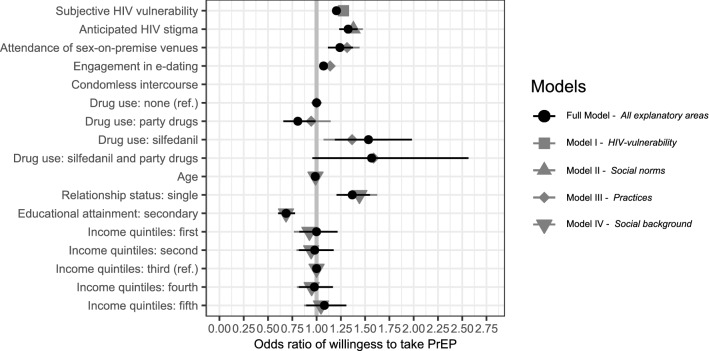
Odds ratio of willingness to take PrEP according to the explanatory areas (condomless intercourse excluded, unstandardized variables). Variables included: subjective HIV-vulnerability, anticipated HIV-stigma, attendance of sex-on-premise venue, engagement in e-dating, drug use, age, relationship status, educational attainment, monthly net household income; All variables are unstandardized, N = 4914

## Data Availability

The data that support the findings of this study are available from the German Bundeszentrale für gesundheitliche Aufklärung (BZgA; “Federal Center for Health Education”). Restrictions apply to the availability of these data, which were used under license for this study. Data is available only from the BZgA.

## Appendix

See Tables 1, 2, 3, 4, 5, and Figs. 3 and 4.

**Table 1:** Overview of indicators

Explanatory area	Indicator	Categories		Original variable		Wording of original variable
Info about PrEP	We would like to know your opinion about the so-called PrEP (abbreviation for Pre-Exposure Prophylaxis. PrEP should not be confused with the Post-Exposure Prophylaxis (PEP), which is taken after a risk situation occurred. HIV-negative people can protect themselves effectively against HIV-infection by regularly taking a preventive HIV-medication (PrEP). However, infections can still occur if the medication is not taken daily. In Europe this preventive method with anti-HIV drugs, has not been approved yet					
Dependent variable	Willingness to take PrEP	0—No 1—Yes		prp5		In principle, would you be prepared to take a medication as a preventive measure to reduce the risk of HIV-infection?
I. HIV-vulnerability	Subjective HIV-vulnerability	0—No risk 10—High risk		sxz2		How high is the risk that you get an infection in the last 12 months?
II. Social norms	Anticipated HIV-stigma	1—Very unlikely 4—Very likely to occur		psy6		Which of the following situations could happen to you if you became infected?
				psy6_1		Friends and acquaintance might think I’ve failed
				psy6_2		Friends would reproach me;
				psy6_3		My family would be disappointed;
				psy6_5		Family and friends would avoid me
III. Practices	Attendance of sex-on-premise venue	1—Never 5—Very frequently		sze1		How often did you visit these places in the last 12 months?
				sze1_4		Darkroom, sex club, public sex party;
				sze1_5		Private sex party;
				sze1_6		Sauna and porn-cinema;
				sze1_7		Outdoor cruising area
	Engagement in e-dating	0—None 5—All of them		sze2		How many sex partners did you meet through mobile apps and the Internet in the last 12 months?
	Condomless intercourse	1—Always (uses condom) 5—Never (uses condom)		sxa11		How often in the last 12 months you used condom during anal intercourse with casual partners?
	Drug use	1—None 2—Party drugs only 3—Viagra only 4—Both		sub4		How often did you use the following substances in the last 12 months?
				sub4_1		Viagra
				sub4_2		Cannabis, Hashish, Marijuana
				sub4_3		MDMA, Ecstasy, Mephedrone
				sub4_4		Methamphetamine, Crystal meth
				sub4_5		Cocaine
				sub4_6		Heroin, Opium, Morphine
				sub4_7		Ketamine, GHB/GBL
IV. Social background	Age	Years		soz2		Please, provide your birth year
	Relationship status	0—Not single 1—Single		bez1		Are you currently engaged in a steady relationship?
	Educational attainment	0—Lower secondary level or below 1—Upper secondary level or higher		ses1		What is your highest professional qualification? Or, if you are still in training, what degree do you aspire to?
	Monthly net household income	1—Lowest quintile 5—Highest quintile		ses5		What is approximately the monthly net income of your entire household?

**Table 2:** Descriptive statistics (*N* = 2948, after listwise deletion)

Variable	Mean/percentage	SD^a^
Willingness to take PrEP	51%	–
Subjective HIV-vulnerability	1.99	2.08
Anticipated HIV-stigma	2.39	0.83
Attendance of sex-on-premise venue	1.58	0.71
Engagement in e-dating	2.96	1.82
Condomless intercourse	2.00	1.38
Drug use		
None (ref.)	79%	–
Use of party drugs only	11%	–
Use of Viagra only	8%	–
Use of both	2%	–
Age	37.14	12.49
Relationship status	56%	–
Educational attainment	63%	–
Monthly net household income		
First quintile	15%	–
Second quintile	21%	–
Third quintile (ref.)	23%	–
Fourth quintile	22%	–
Fifth quintile	19%	–

^a^If applicable

**Table 3:** Comparison of descriptive statistics between respondents included in the sample and respondents who have insufficient knowledge of PrEP

Variable	Sufficient knowledge (included in analysis)			Insufficient knowledge of PrEP		
	N	Mean/%	SD	N	Mean/%	SD
Subjective HIV-vulnerability	5950	1.36	1.66	5646	1.41	1.86
Anticipated HIV-stigma	5760	2.38	0.78	5426	2.37	0.84
Attendance of sex-on-premise venue*	5957	1.38	0.57	5648	1.42	0.64
Engagement in e-dating	5953	2.36	2.04	5635	2.28	2.04
Condomless intercourse*	3666	1.78	1.24	3372	1.99	1.38
Drug use	5830			5484		
None (ref.)		83%	–		82.6%	–
Use of party drugs only		9.7%	–		9.8%	–
Use of Viagra only		6.1%	–		6.1%	–
Use of both		1.2%	–		1.6%	–
Age	5961	37.3	12.95	5652	37.4	12.91
Relationship status*	5956	53%	–	5645	49%	–
Educational attainment*	5904	65%	–	5583	63%	–
Monthly net household income*	5503			5179		
First quintile		17.2%	–		16.6%	–
Second quintile		21.5%	–		20.5%	–
Third quintile (ref.)		22.4%	–		21.7%	–
Fourth quintile		22.0%	–		21.8%	–
Fifth quintile		16.9%	–		19.3%	–

*Significant difference detected

**Table 4:** Detailed reports of significant differences for continuous independent variables in Table 3

	T-test	p-value
Attendance of sex-on-premise venue	3.497	< 0.001
Condomless intercourse	6.498	< 0.001

Group A: sufficient knowledge (included in analysis); Group B: insufficient knowledge of PrEP (excluded from analysis)

**Table 5:** Detailed reports of significant differences for categorical independent variables in Table 3

	Chi-square	p-value	Cramer`s V
Relationship status	16.399	< 0.001	0.038
Educational attainment	5.978	0.015	0.023
Monthly net household income	11.185	0.025	0.032

Group A: Sufficient knowledge (included in analysis); Group B: Insufficient knowledge of PrEP (excluded from analysis)

## Abbreviations

ART: Antiretroviral therapy
CDC: Centers for Disease Control and Prevention
CI: Confidence interval
GHB: Gamma hydroxybutrate
MSM: Men who have sex with men
OR: Odd ratio
PrEP: Pre-exposure prophylaxis
STI: Sexually transmitted infection
WHO: World Health Organization
